# Achieving net-zero agriculture in Africa: perspective on policies, challenges, and opportunities

**DOI:** 10.1007/s11625-025-01666-y

**Published:** 2025-03-17

**Authors:** Tersur Theophilus Akpensuen, M. Jordana Rivero

**Affiliations:** 1 Net-Zero and Resilient Farming, Rothamsted Research, North Wyke, Okehampton, EX20 2SB UK; 2 Faculty of Agriculture, University of Jos, Jos, 930001 Nigeria

**Keywords:** Continental, Regional, Economic communities, Agricultural emissions, Frameworks, Agriculture, Forestry and other land use

## Abstract

Africa, with 55 Member States and over 1 billion people, is projected to nearly double its population to 2.5 billion by 2050, presenting both opportunities and challenges for sustainable development. Agriculture employs 65% of the labour force and contributes 32% to gross domestic product. The aim of this perspective is to highlight the challenges and opportunities of achieving net-zero agriculture in Africa while proffering appropriate recommendations. The primary issues are how extreme weather events affect food security and how to cut emissions from livestock farming as well as rice cultivation and fertilizer usage alongside evaluating current policies that support climate-smart agricultural practices. Africa needs to investigate how its expanding young population along with research and innovation can advance the move towards net-zero agricultural practices. Challenges of insufficient data availability together with ineffective policy enforcement, financial barriers, and limited awareness, decreasing precipitation levels coupled with regional conflicts and population migration hinder progress in achieving net-zero agriculture on the continent. However, Africa possesses substantial opportunities through its extensive arable land combined with its youthful workforce and renewable energy capabilities. Africa needs to obtain climate funds and strengthen regional partnerships while enhancing climate information services and creating inclusive and gender-responsive policies to address these issues. Investing in innovative technologies alongside renewable energy sources and crops resistant to climate change stands as an essential strategy. The implementation of early warning systems along with the development of alternative livelihoods will enhance efforts towards sustainable management of climate-induced migration. Africa will develop a climate-resilient agricultural system by confronting existing challenges while capitalizing on emerging opportunities.

**Supplementary Information:**

The online version contains supplementary material available at 10.1007/s11625-025-01666-y.

## Introduction

Africa comprises 55 Member States with a population of over 1 billion people, all of whom are members of the African Union. It is home to the world’s youngest and fastest-growing population, with its population expected to nearly double to 2.5 billion by 2050; the continent has myriad opportunities to harness its rich natural resources and abundant human potential to increase economic growth and prosperity not only in Africa, but also around the globe (Kuyoro et al. 2023). Compared with 2010, population growth and changes in dietary requirements are projected to triple the demand for food by 2050 (Van Ittersum et al. 2016). The entire continent has already experienced an increase in emissions, mostly attributable to deforestation, of which agriculture is the main cause (Curtis et al. 2018; Carter et al. 2017). The continent is the eleventh largest economy in the world with a nominal gross domestic product (GDP) of approximately US$ 2.3 trillion (ACCS 2020) and is the second largest continent after Asia (Middleton et al. 2024)*.*

Agriculture is a cornerstone of Africa’s economy, employing about 65% of the labour force and contributing 32% to the continent’s GDP, though productivity remains relatively low (Goedde et al. 2019). Growth in agricultural production has historically relied on land expansion in Africa and Latin America (Tilman et al. 2011; Herrero et al. 2016) and could be further boosted through intensification methods like improved inputs, practices, and crop varieties (Hickman et al. 2011). However, these activities, along with land clearing, soil cultivation, and fertilizer use, contribute significantly to greenhouse gas (GHG) emissions, primarily from agriculture, forestry, and other land use (AFOLU) (Tilman et al. 2011). According to Boko et al. (2007), Africa faces severe climate challenges, including droughts, floods, deforestation, and rising sea levels, which threaten agricultural productivity and food security, and will potentially reduce rain-fed yields by up to 50% by 2050. Agricultural practices also impact the environment through high water use, biodiversity loss, and soil degradation (Zhou et al. 2021). Key sources of GHG emissions in agriculture include energy use, land use, livestock production, and rice cultivation (Chalise and Naranpanawa 2016), accounting for 13–21% of global anthropogenic GHG emissions between 2010 and 2019 (Nabuurs et al. 2022). In Africa, nitrous oxide (N_2_O) emissions account for 42–71% of the continent’s anthropogenic N_2_O emissions and 6–11% of global N_2_O emissions, with projections suggesting a potential doubling by 2050 (Hickman et al. 2011). The continent also produces 343.2 million tons carbon dioxide equivalent (CO_2_e) of enteric methane (CH_4_) emissions from ruminants, compared to 732.5, 206.6, 179.2, 460.4, and 67.6 million tonnes CO_2_e from Asia, Europe, North America, and Oceania, respectively (FAOSTAT 2020). Addressing these challenges is critical for sustainable development and food security in Africa.

The goal of reducing GHG emissions is to limit the accumulation of these gases in the atmosphere, which leads to increased radiative forcing (global warming potential). Reduced global warming from agriculture can be achieved by increasing the storage of soil organic carbon, and reducing CH_4_ and N_2_O emissions while increasing productivity. Agriculture plays a dual role as both a sink and a source of GHG emissions, with the potential to sequester an estimated 4% of global GHG emissions by the end of the century (Henderson et al. 2022). However, the current understanding of GHG emissions in sub-Saharan Africa (SSA) is minimal compared with the continent’s potential as both a GHG sink and a source (Kim et al. 2016a, b; Boateng et al. 2017), and the limited data and information on agricultural production in Africa make it difficult to quantify the continent contributions of GHGs to total global emission. There is limited discussion on the effectiveness of climate-smart farming practices in reducing emissions and enhancing resilience in African contexts. Additionally, the role of smallholders, who primarily dominate agricultural activities, and the importance of Indigenous knowledge in reducing GHG emissions across the continent are not well understood. Moreover, climate financing mechanisms are lacking, and there are no robust systems in place for monitoring, reporting, and verifying climate-related activities as well as implementation of climate-related policies. There is a societal urgency to transition towards a more sustainable food industry, with reduced GHG emissions and increased carbon sequestration, while also protecting and enhancing biodiversity and soil health (Pawlett et al. 2021). Achieving balanced economic growth without compromising the environment is key to sustainable development, and agricultural management practices/policies are being implemented to meet the target of environmental sustainability.

Net zero involves reducing carbon emissions to minimal levels, with any remaining emissions being absorbed and stored by natural processes and other CO_2_ removal methods, resulting in no net increase in atmospheric CO_2_. This concept is central to achieving the Paris Agreement goals, which aim to limit global warming to below 2 °C by the end of this century, with efforts to further cap the increase at 1.5 °C above preindustrial levels. The Paris Agreement, adopted by 195 countries during the COP21 conference, includes a key directive in its fourth article: “*countries should strive to balance human-caused greenhouse gas emissions and their removal by the second half of the twenty-first century*” to meet this target, emissions must be cut by 45% and achieve net zero by 2050 (IPCC 2018). China, the USA, India, and the European Union, representing approximately 88% of global emissions, and over 9000 companies, more than 1000 cities, 1000 educational institutions, and 600 financial institutions (UNEP 2023) have pledged to reach net-zero emissions. These entities have vowed to take immediate, rigorous action to halve global emissions by 2030. Given the substantial contribution of agriculture to emissions, many of these countries and organizations have launched initiatives targeting net-zero agriculture by 2050.

While many African countries may not have specific programmes targeting net-zero agriculture, there are programme and policies to promote climate-smart and sustainable agricultural practices without explicitly identifying net-zero agriculture programmes. African Union (AU), the African Development Bank (AfDB), and the United Nations Food and Agriculture Organization (FAO) are supporting many African nations to incorporate climate-smart resilience and low-carbon strategies into their agricultural policies. All its continental and regional climate change mitigation policies and frameworks aim to achieve net-zero emissions as Africa is a signatory to the Paris Agreement. In this regard, questions relate to: How does the African continent balances the need for increased agricultural production with the environmental impacts of land clearing, deforestation, and GHG emissions? To what extent do extreme climate events (e.g. droughts, floods, rising sea levels) affect agricultural production and food security in different regions of Africa? How can Africa reduce its GHG emissions from agriculture, particularly from livestock production, rice cultivation, and nitrogen fertilizer use? What are the projected impacts of climate change on rain-fed agriculture in Africa by 2050? What are the policies and frameworks currently in place in Africa to promote climate-smart and sustainable agriculture? How effective are they? What are the social and cultural challenges in transitioning to net-zero agriculture in Africa? What are the current gaps in data and research on GHG emissions from agriculture in sub-Saharan Africa? How can Africa harness its young and growing population to drive innovation and productivity in agriculture? The overall big question is how to address the above question among others in achieving net-zero agriculture in Africa through effect policies. Policymakers, government officials, researchers, academics, agricultural and environmental organisations, private sectors, agribusiness leaders, development agencies, financial institutions, agricultural cooperatives, and young professionals can play a critical role in achieving net-zero agriculture and are the principal target audiences for the manuscript. This manuscript examines some of the policies targeting net-zero agriculture, identifies the challenges Africa faces in achieving net-zero agriculture, and explores the opportunities available to balance and sink emissions from agriculture on the continent.

## Global and regional total greenhouse gas emission trends

**Fig. 1:**
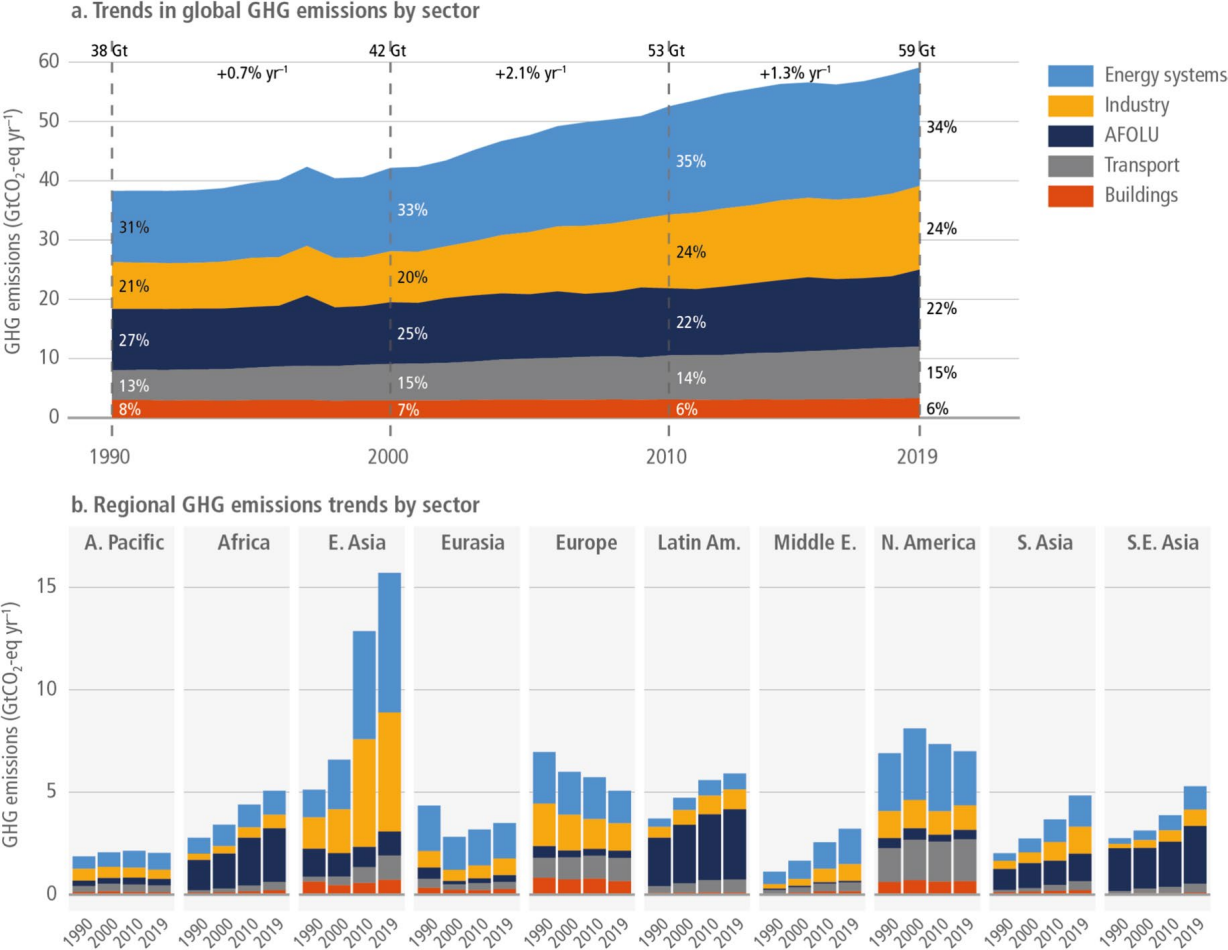
Global and regional GHG emission trends for all sectors. Panel **a** shows total global anthropogenic GHG emissions divided into major sectors. Panel **b** shows regional emission trends in the years 1990, 2000, 2010, and 2019 (Parmesan et al. 2022)

Agriculture is a significant contributor to GHG emissions and thus plays a crucial role in climate change (Lynch et al. 2021). This contribution involves various activities and practices that release CO_2_, CH_4_, and N_2_O. These gases have different global warming potentials, with methane and nitrous oxide being particularly potent compared with CO_2_ (Tanaka et al. 2009). According to data from Parmesan et al. (2022), the agriculture sector contributes the third highest percentage of GHG emissions, accounting for approximately 22%, 11.9 ± 4.4 GtCO_2_-eq year^–1^ on average over the period 2010–2019 (Fig. 1a). The data indicates that the contribution of emissions from AFOLU to total emissions decreased by 5% over three decades (1990–2019), whereas emissions from all other sectors increased, except for emissions from buildings.

According to Parmesan et al. (2022), regional trends in total GHG emissions show that the African continent is among the lowest emitters, with East Asia being the highest emitter (Fig. 1b). The data also indicate that North America and Europe are among the top emitters of GHGs. Historically, Africa’s contribution to global greenhouse gas emissions has been relatively small, estimated at approximately 3–6% (Wang et al. 2024). However, according to the climate change vulnerability Index study by Sarkodie and Adams (2018), the top 10 countries most vulnerable to climate change are all from SSA. Africa’s contribution to total GHG emissions has slowly, but steadily increased from approximately 3 GtCO_2_eq year^−1^ in 1990 to approximately 5 GtCO_2_eq year^−1^ in 2019. Compared with 1990, Europe, North America, and Eurasia are the regions that showed a reduction in GHG emissions in 2019 (Fig. 1b).

## Global and regional total greenhouse gas emissions and removal trends from agriculture, forestry, and other land use (AFOLU)

**Fig. 2:**
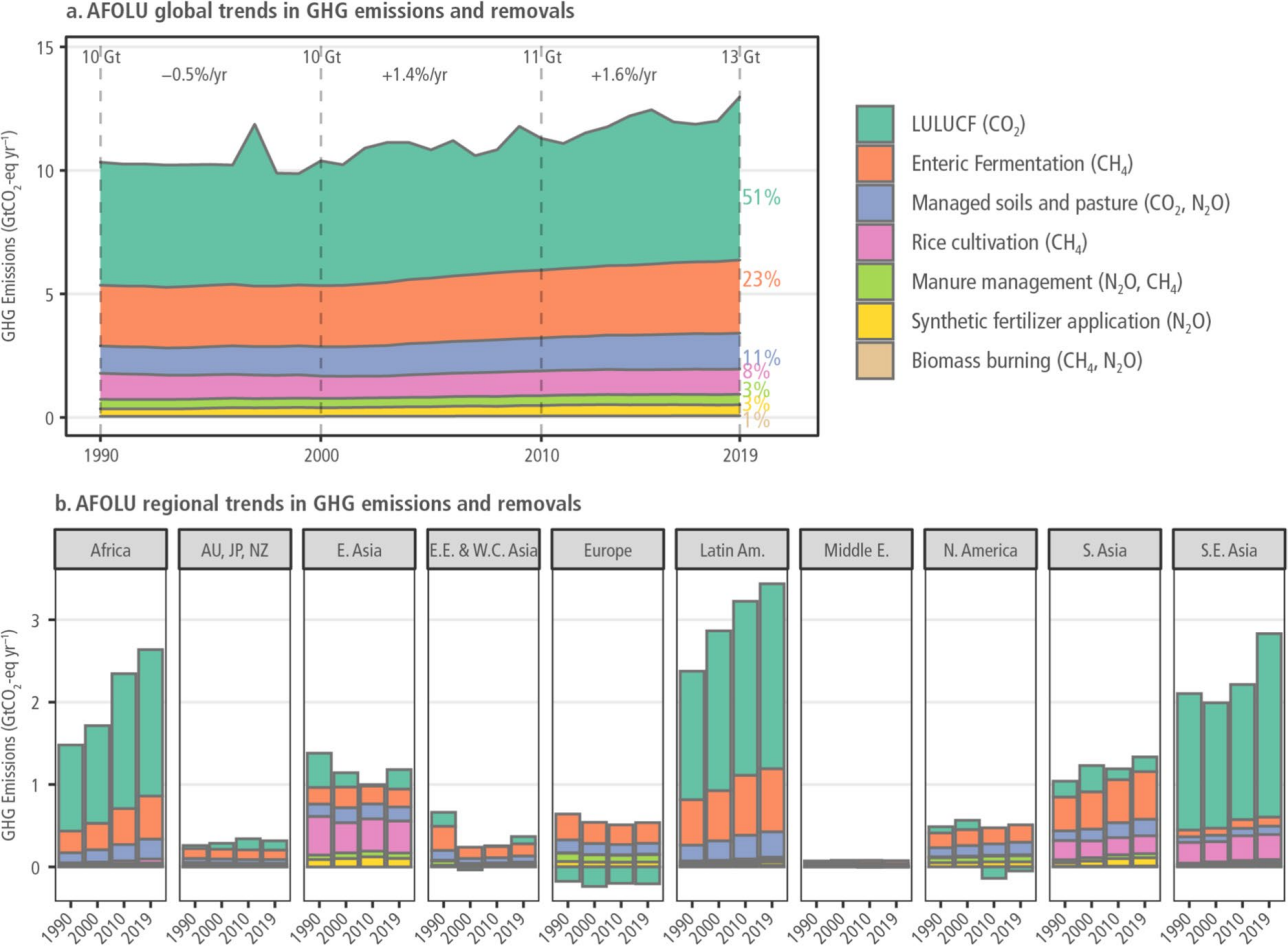
The global (**a**) and regional (**b**) greenhouse gas (GHG) emission and removal trends from Agriculture, forestry and other land use (AFOLU) in 1990, 2000, 2010 in 1990, 2000, 2010 (Cissé et al. 2022)

Data from the FAO presented by Cissé et al. (2022) revealed the total global and regional trends of net anthropogenic GHG emissions from AFOLU (Fig. 2). Land use, land use change, and forestry (LULUCF) contributed the most emissions, followed by enteric CH_4_ emissions with 51% and 23%, respectively, from 1990 to 2019 (Fig. 2a). The emissions from LULUCF and enteric CH_4_ emissions have substantially increased over the past three decades (1990–2019).

The contribution of AFOLU to total emissions varies regionally, with the highest contribution in Latin America and the Caribbean with 58% and the lowest in Europe and North America with 7% at the regional scale (Fig. 2b). Africa is the third highest emitter of GHG from AFOLU increasing from about 1.5 GtCO_2_-eq year^–1^ in 1990 to 2.6 GtCO_2_-eq year^–1^ in 2019 resulting in approximately 42% increase during this period. Since 2000, Asia has had the largest share (37%) of emissions from enteric fermentation and manure management (Smith 2014), while Africa had the fastest growth rate (Cissé et al. 2022). Asia is responsible for most of the rice cultivation GHG emissions, which have been increasing (Jia et al. 2019). All the databases indicate significant emission growth in Africa since 1990, with the region showing the second-highest increase in emissions from enteric fermentation and rice cultivation since 2010.

In terms of agricultural emission removal, while the fewest emitters are making positive progress, there is no progress from the largest AFOLU emitters (Latin America, Southeast Asia, and Africa) (Fig. 2b). The food system of SSA (crop production and livestock farming) is responsible for approximately 40–60% of the annual emissions of GHG within the continent (Omotoso and Omotayo 2024). These results showed that AFOLU are major contributors to total GHG emissions from Africa and there is an urgent need to speed up sustainable agricultural practices that will lead to net zero.

**Fig. 3:**
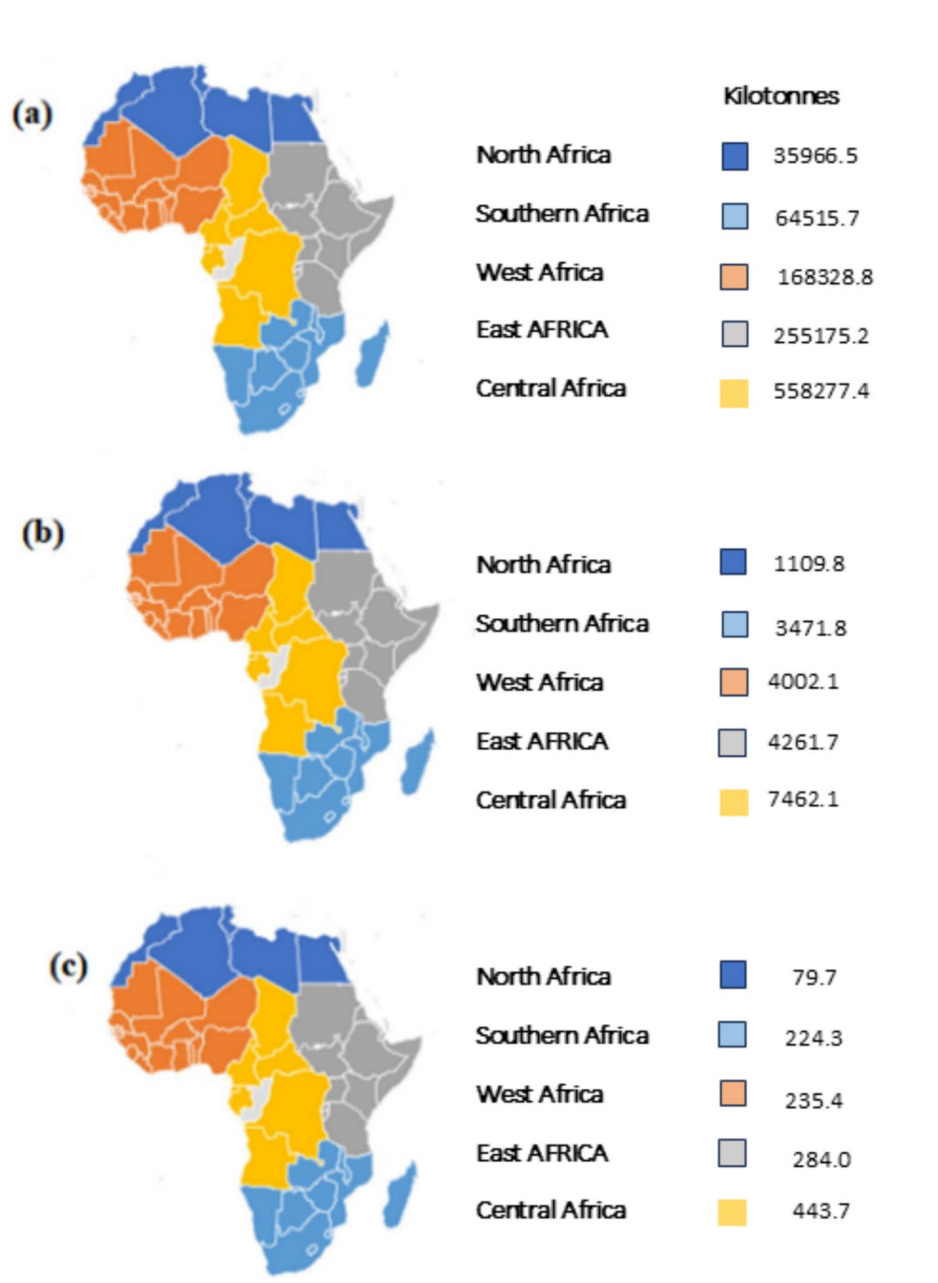
Spatial distributions of emission scores for **a** CO_2_, **b** CH_4_, and **c** N_2_O from agricultural land in Africa from 1990 to 2019 (Epule et al. 2022)

In terms of the distributions of CO_2,_ CH_4_, and N_2_O from agriculture presented by Epule et al. (2022), middle/central Africa (CA) recorded the highest CO_2_ with a score of 40 to the maximum score of 40%, whereas the lowest scores were recorded in North Africa and South African regions with a score 10% (Fig. 3a). An analysis of FAO data by Tongwane and Moeletsi (2018) was also historically consistent with greater CO_2_ emissions from CA. This finding is not surprising due to the level of deforestation for mining activities, crop cultivation, and other land uses especially in the DR Congo.

In terms of CH_4_ scores, Epule et al. (2022) reported East Africa as the largest contributor with the highest score of 30 to the maximum score of 30%, whereas Southern Africa recorded the lowest score of 15% (Fig. 3b). Furthermore, Tongwane and Moeletsi (2018) reported a consistent trend over time as enteric CH_4_ emissions were higher from East Africa. The greatest CH_4_ intensity was in Eastern Africa with an average of 233 g CH_4_ kg^−1^ of milk, but the intraregional variation was smaller than that in Western Africa (SD = 110 g CH_4_ kg^−1^ of milk) (Dolecheck and Bewley 2018). The region is known to be home to many domestic and wild ruminants. This region is characterized by mainly smallholder farmers, who experience various challenges including problems with overgrazing, a lack of infrastructure (very few milk collection locations and product manufacturers), little access to credit, and difficulty transferring technical knowledge and skills to farmers (Dolecheck and Bewley 2018). In terms of N_2_O scores, East Africa records the highest possible score of 30 while Southern Africa records the lowest possible score of 10 (Fig. 3c). The regions of Central, West, and North Africa all had record scores of 15.

## Global projections of agriculture, land use, and forestry emissions in 2050

**Fig. 4:**
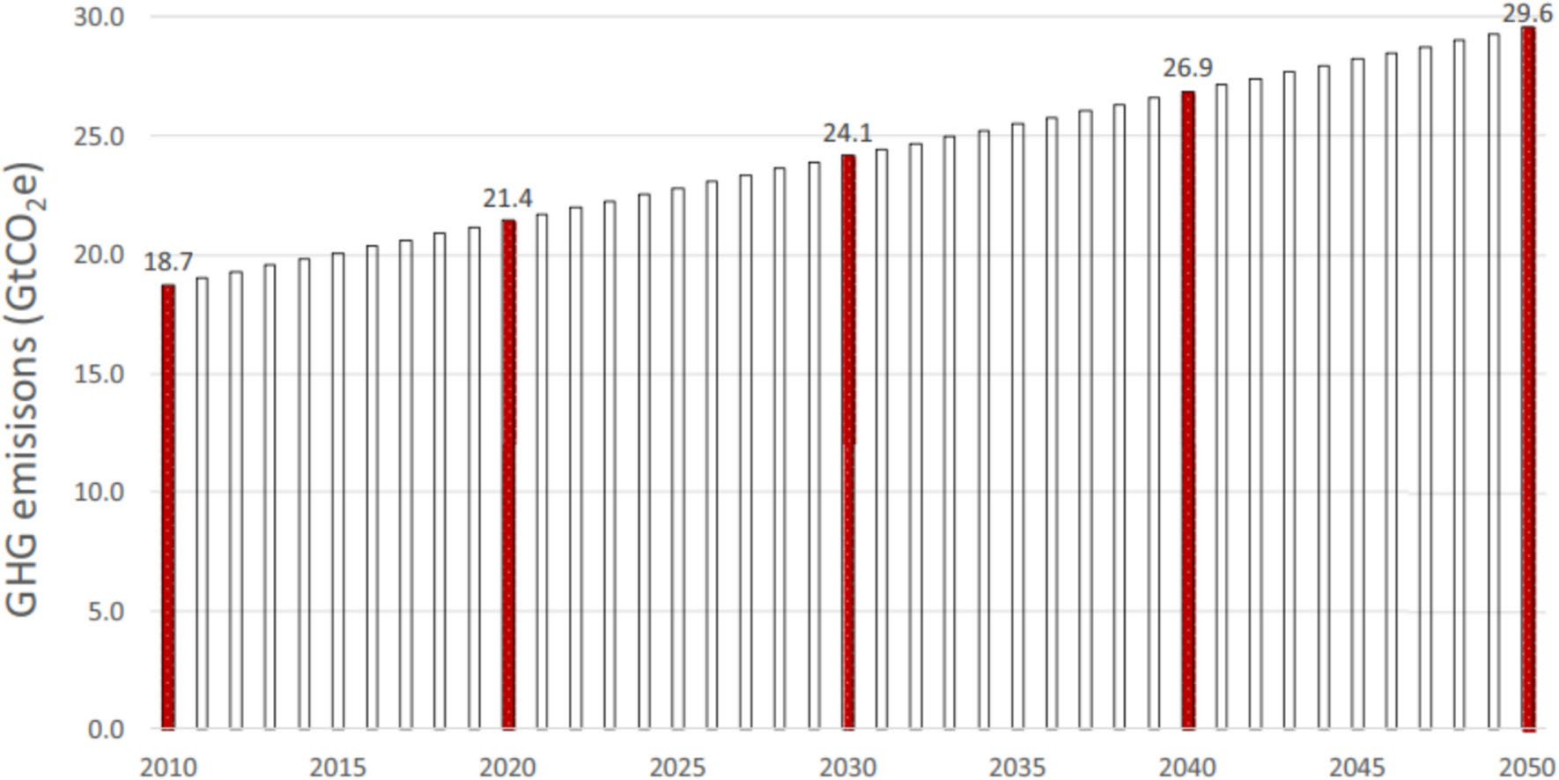
Global food system emissions in estimated global food system emissions from 2010 to 2050 (Costa Jr et al. 2022)

**Fig. 5:**
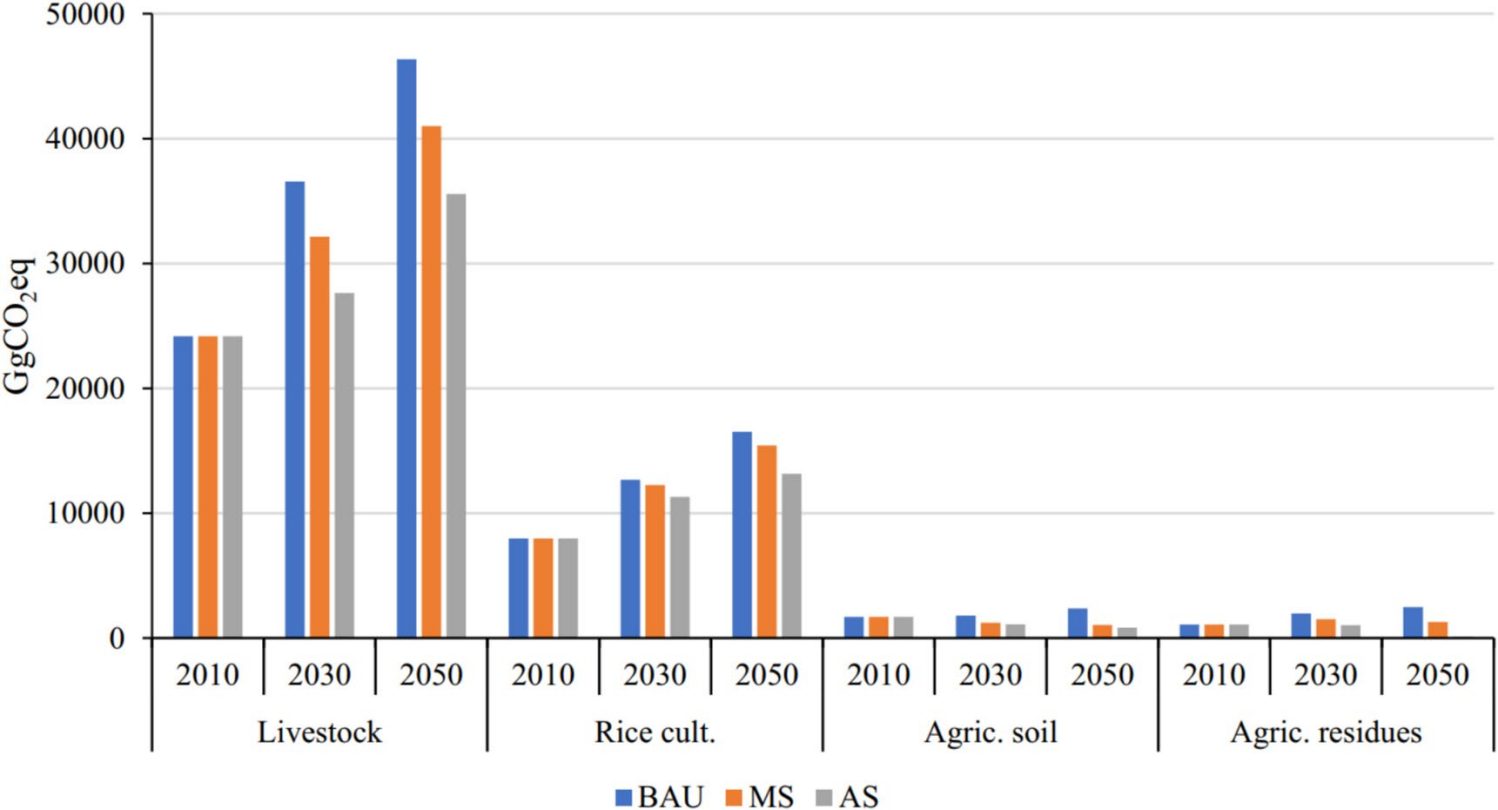
Breakdown of GHG emissions by source category in 2010 and 2030, and from business as usual (BAU), moderate scenarios (MS), and aggressive (AG) 2050 (Dioha and Kumar 2020)

**Fig. 6:**
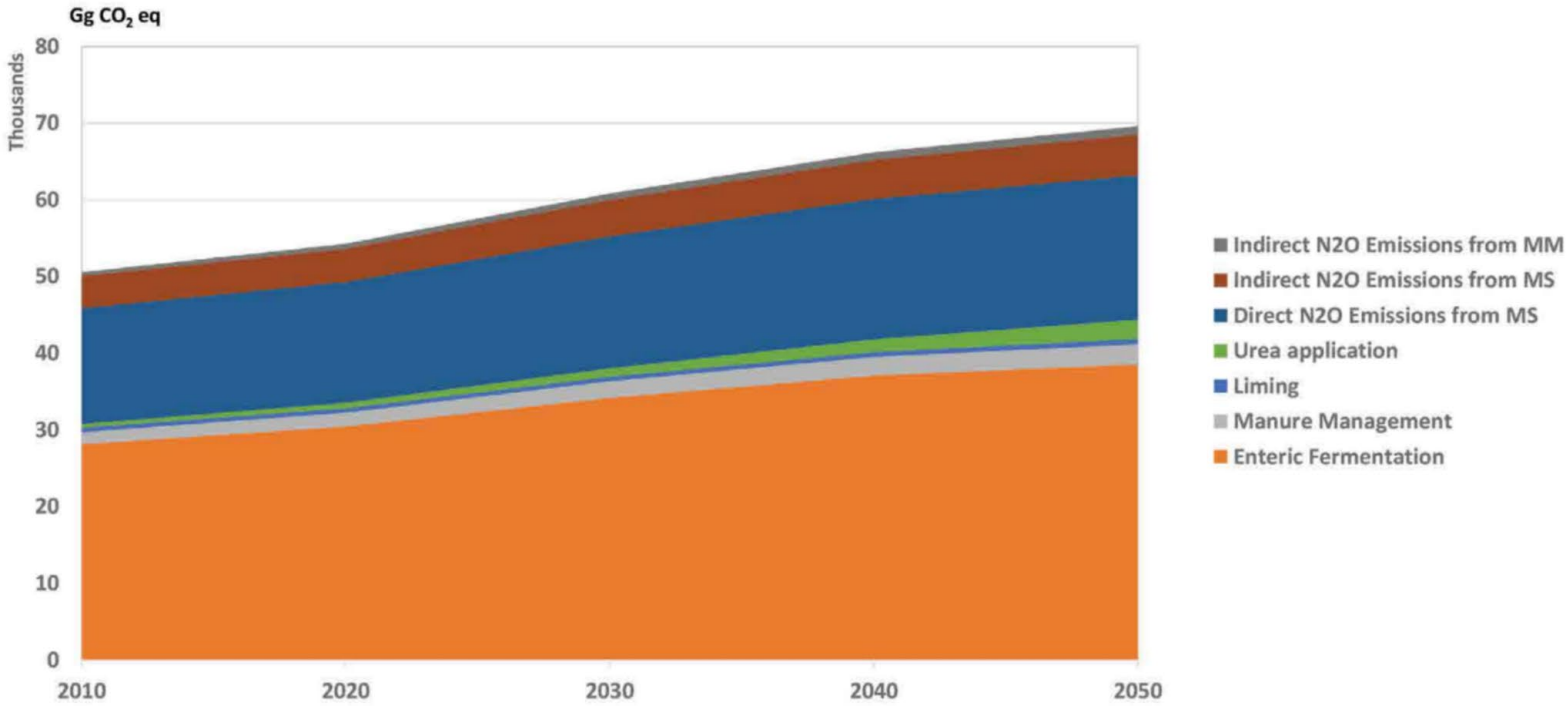
Contribution of the various categories to the agricultural baseline in South Africa (MM = manure management; MS = managed soils) (DEA 2016)

Costa Jr et al. (2022) reported that global food systems emitted between 18.7 and 21.4 GtCO_2_eq year^−1^ from 2010 to 2020 (Fig. 4). They further projected that, under current average production practices, GHG emissions would rise to 29.6 GtCO_2_eq year^−1^ by 2050 to meet the expected increase in food production. In the African region, taking Nigeria as an example, Dioha and Kumar (2020) projected a rise from 24,172 GgCO_2_eq in 2010 to 46,349 GgCO_2_eq by 2050 in the business-as-usual (BAU) scenario, as livestock remain the largest source of GHG emissions. Compared with those in 2010, enteric CH_4_ emissions from livestock are projected to increase by 52.5% in 2030 and 92.4% in 2050. Those from rice cultivation are also expected to rise significantly by 2050 (Fig. 5). Similarly, according to DEA (2016), enteric fermentation is the largest contributor to agricultural emissions, accounting for approximately 56% and showing a 3% increase from 2010 to 2050 in South Africa (Fig. 6). The general increase in GHG emissions across various sources is driven primarily by the projected increase in livestock numbers, nitrogen fertilizer use, and the expansion of cultivated crop areas. Therefore, implementing low-emission practices in livestock and other agricultural sectors on a large scale is essential for achieving significant reductions in emissions and moving towards net zero.

## Tackling agricultural emission on the continent of the Africa

Addressing agricultural emissions in Africa is a critical component of the continent’s strategy to combat climate change while ensuring food security and sustainable development. Agriculture is not only a major economic activity in Africa, supporting the livelihoods of millions, but also a significant source of GHG emissions, primarily through enteric fermentation in livestock, manure management, rice cultivation, and soil fertilization. The unique challenges faced by African countries, including population growth, reliance on traditional farming practices, and the impacts of climate variability, necessitate tailored and effective policy interventions. The development and implementation of policies that promote sustainable agricultural practices, increase productivity, and reduce emissions are imperative. These policies must balance the urgent need for climate action with the goals of poverty alleviation and economic development, ensuring a resilient and prosperous future for the continent.

Numerous policies, frameworks, and action plans have been developed at the continental and regional levels to address emissions from agriculture. These frameworks and policies should be implemented to mitigate the impact of climate change. Here, we focused more on those policies and frameworks related to agriculture. Importantly, this article does not provide a complete history of agriculture-related policies at the continental and regional levels in Africa. This article focuses on policies from 2000 and onwards. One article cannot encompass regional and continental policies over several decades. This article also did not focus on international organisations and other countries role in agricultural transformation. In this work, we focused on three main areas of programmes/policies enacted by political leaders to reduce the impact of climate change on agriculture. These included the climate for development in Africa, and continental and regional policies/frameworks. Importantly, the authors did not find any policies specifically targeting net-zero agriculture in Africa. Most of the programmes aimed to reduce emissions in general rather than focusing solely on agriculture. These policies/frameworks are broadly classified into three categories: climate development for Africa, climate change negotiation structure, and continental and regional level policies.

### Climate for development in Africa

**Fig. 7:**
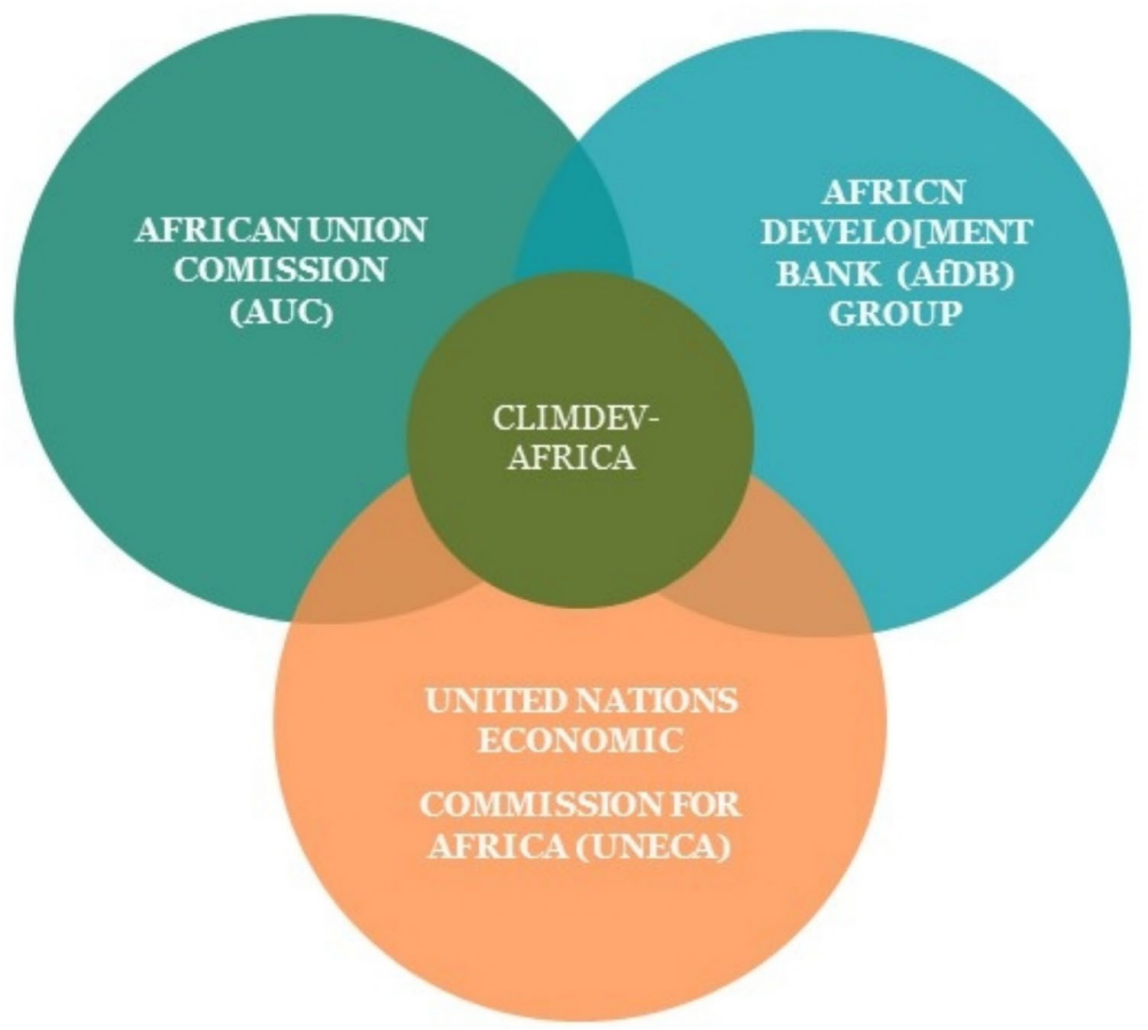
Climate for development in Africa (Cooke 2022)

According to Cooke (2022), ClimDev-Africa is an initiative established by the African Union Commission (AUC), the United Nations Economic Commission for Africa (UNECA), and the African Development Bank (AfDB). Figure 7 illustrates the relationships between ClimDev-Africa and these three organizations. Mandated at the highest level by the AU Summit of Heads of State and Government, the programme aims to create a robust foundation for Africa’s climate change response. In addition to the AUC–UNECA–AfDB partnership, ClimDev-Africa collaborates with various African and international institutions and partners specializing in climate and development. Its primary objectives are to (i) address climate information gaps by enhancing climate information services, (ii) promote sustainable development, (iii) build resilience among vulnerable communities, and facilitate analyses that support effective policies and decision-making at all levels, (iv) develop early warning systems, climate data collection and dissemination platforms, (v) strengthen the capacity of African institutions and stakeholders to integrate climate information into development planning, (vi) influence regional and global climate policies to reflect African priorities and needs, and (vii) generate knowledge and innovative solutions to address climate change. The programme has achievements in contribution to national adaption plans in several African countries, provides forecasts that assist farmers in planning planting and harvesting, and fosters collaboration among countries in knowledge sharing and coordination responses to climate challenges.

### Climate change negotiation structure

Africa has a three-tiered climate change negotiation structure in the multilateral climate change negotiations under the United Nations Framework Convention on Climate Change (UNFCCC), as supplemental material (Table S1). These UNFCCC negotiations are marked by asymmetries in negotiating capacity and bargaining power among participating countries (Bhandary 2017). It is a strategic approach designed to amplify the continent’s voice, ensure coherence, and maximize its influence in global climate discussions. This structure reflects the continent’s recognition of the disproportionate impacts of climate change on African nations and the need for a unified and coordinated approach to address these challenges. Africa’s role in the UNFCCC is crucial, given the region’s vast pristine tropical rainforests, abundant renewable energy sources, and the world’s youngest population (Kuyper et al. 2018).

### Continental and regional policies and frameworks

The aims and time frames of the continental and regional-level policies are presented in supplementary materials Table S2 and Table S3. The set of policies outlined for Africa represents a comprehensive and interconnected approach to achieve net zero emissions across the continent. These policies are designed to address the multifaceted challenges of climate change by promoting sustainable development, enhancing climate resilience, and fostering innovation. At the core of these policies is Agenda 2063, which serves as a strategic blueprint for transforming Africa into a global powerhouse, emphasizing the importance of climate-smart development and self-reliance. This overarching vision is supported by various sector-specific strategies, such as sustainable forest management, agricultural development, and climate adaptation, which collectively aim to reduce emissions and build resilience across different sectors. The policies also emphasize the importance of climate finance and capacity building, recognizing that achieving net zero will require significant investments and enhanced capabilities across the continent. Furthermore, initiatives such as the Great Green Wall and the African Forest Landscape Restoration Initiative illustrate a strong commitment to land restoration and carbon sequestration, which are key components in the fight against climate change.

**Fig. 8:**
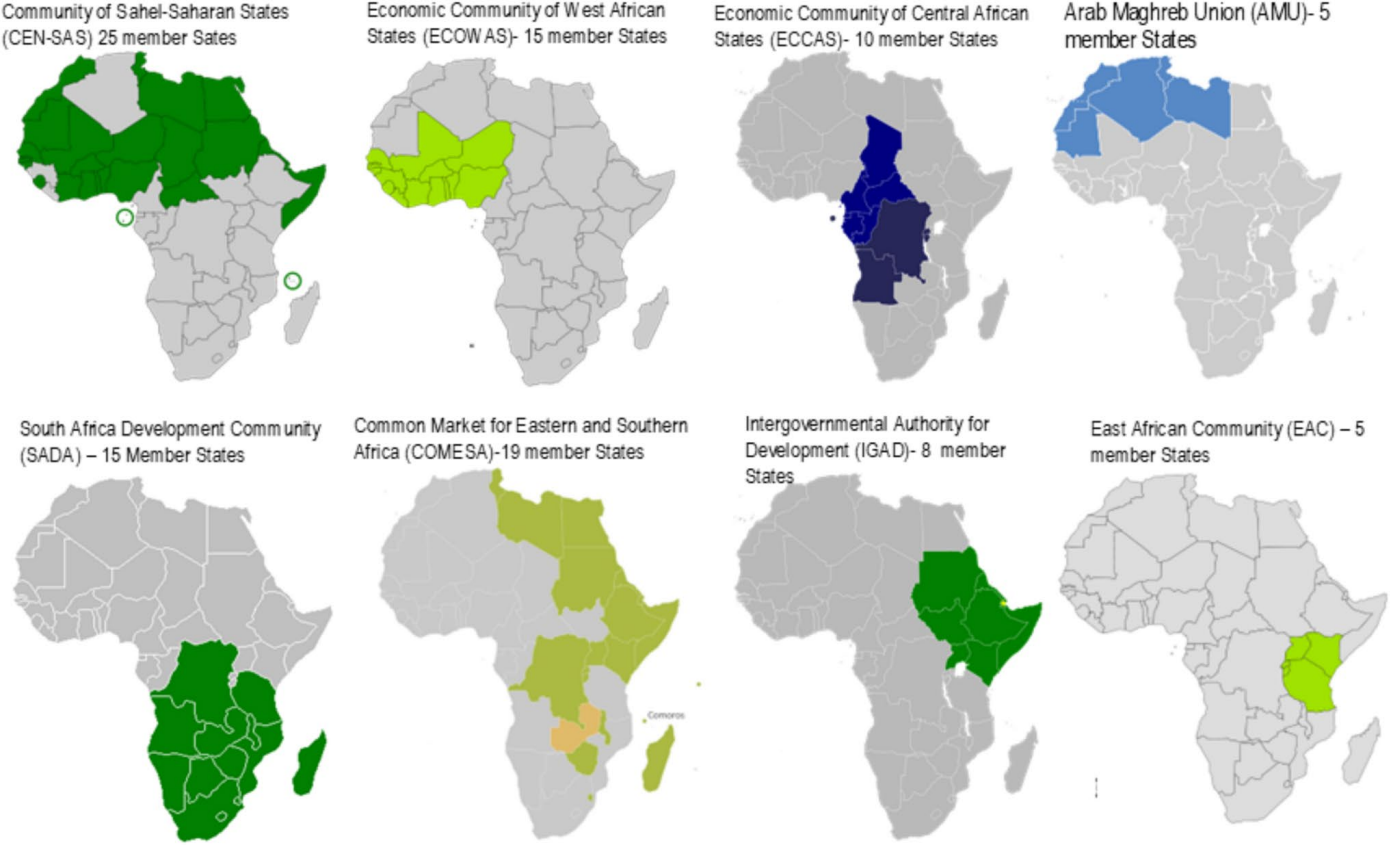
Map of Africa showing the membership of regional economic communities

Figure 8 presents a map of Africa showing the membership of regional economic communities. Regional climate centres are fundamental components that support the achievement of several important climate initiatives at the continental level and are designed to improve the provision and use of appropriate climate information to promote planning for sustainable development in Africa. The regional economic communities include the East Africa Community (EAC), the Economic Community of West African States (ECOWAS), the South African Development Community (SADC), the Common Market for East and Southern Africa (COMESA), the Economic Community of Central African States (ECCAS), and Intergovernmental Authority on Development (IGAD). The policies of the Regional Climate Centres in Africa align with the continent’s net-zero goals by establishing a coordinated and multifaceted approach to climate change adaptation, mitigation, and resilience building. These policies reflect the diverse climate challenges faced across Africa’s regions, and they emphasize the importance of collective action, regional solidarity, and the integration of climate considerations into all sectors of development. The policies prioritize coordinated regional strategies, ensuring that climate actions are harmonized across borders. This collective approach is crucial for addressing shared climate risks, such as desertification, extreme weather events, and water scarcity. Many of these policies, such as those from EAC, SADC, and ECOWAS, aim to build resilience against climate impacts while simultaneously promoting low-carbon development pathways. This dual focus supports the transition to net zero by reducing greenhouse gas emissions while enhancing adaptive capacities. Strategies such as the COMESA Climate Change Strategy emphasize unlocking resources and enhancing technical capacities, which are essential for implementing effective policies such as the ECCAS Action Plan, which incorporates gender-responsive approaches and sustainable development principles, ensuring that the transition to net zero is inclusive and equitable.

## Challenges of achieving net zero in Africa

### Limited data availability

The current understanding of GHG emissions in SSA is notably limited, despite the continent’s significant potential to both absorb and produce GHGs (Kim et al. 2016a, b; Boateng et al. 2017). This lack of understanding is due to various factors, such as incomplete data, inadequate monitoring systems, and the complexity of emission sources and absorbers in the region. Sub-Saharan Africa faces challenges in collecting and analysing GHG emissions data because many countries in the region lack the necessary financial and technical resources to set up effective monitoring systems. As a result, the data are often sporadic and outdated, making accurate assessment of current emissions levels difficult. Additionally, the diverse ecosystems in SSA, from dense tropical forests to expansive savannas, require specific monitoring approaches, further complicating data collection efforts.

### Decreasing precipitation

Achieving net-zero agriculture in Africa is a significant challenge, worsened by declining precipitation patterns across the continent. Agriculture is a primary source of income for millions of Africans and a crucial part of the continent’s economy and food security. However, decreasing precipitation due to climate change poses significant risks to agricultural productivity and sustainability (Saleem et al. 2024) complicating efforts to achieve net-zero emissions in the sector. Decreased precipitation directly impacts crop growth and yields. Water scarcity limits the ability of crops to develop, reducing overall productivity. Crops such as maize, sorghum, and millet, which are staple crops in many African countries, are particularly vulnerable to reduced rainfall (Derbile et al. 2022). Lower yields not only threaten food security, but also increase the pressure to convert more land for agricultural use. This has the potential to result in deforestation and increased greenhouse gas emissions, leading to increased emission intensity.

According to Boko et al. (2007), rain-fed agricultural productivity could decrease by as much as 50% by 2050 in certain countries. The Intergovernmental Panel on Climate Change (IPCC) predicts that wheat may disappear from Africa by 2080, and that maize productivity will significantly decrease in southern Africa. There will be decreases in precipitation in northern and southwestern South Africa, whereas the Ethiopian Highlands are likely to experience increases in rainfall and extreme rainfall by the end of the twenty-first century (Niang et al. 2014). Decreasing rainfall leads to poor harvests, which can prompt farmers to migrate to cities in search of better livelihoods. Arid and semiarid lands are set to increase, with severe consequences for livelihoods, poverty eradication, and meeting the SDG and the Agenda 2063 deadlines (UNEP 2022). Dry conditions lead to reduced organic matter, soil compaction, and erosion, reducing soil fertility due to reduced microbial activity (Szejgis et al. 2024). Poor soil health further decreases agricultural productivity and resilience (Stewart 2016; Baggaley et al. 2021), making it more difficult for farmers to maintain sustainable practices to achieve net-zero emissions. As natural rainfall becomes less reliable, the demand for irrigation increases. However, many African countries face water infrastructure and resource limitations, making large-scale irrigation unsustainable. Overextraction of water for irrigation can deplete water tables and reduce water availability for other uses, creating a cycle of scarcity that hinders sustainable agricultural practices.

### Conflicts and migration

Conflicts disrupt agricultural activities, displace populations, and create instability, whereas migration exacerbates resource pressures and complicates sustainable land management. Together, these factors impede efforts to reduce GHG emissions and promote sustainable agricultural practices. Conflict accounts for at least 75% of the population displacement in Africa (Osman and Abebe 2023), affecting parts of Africa, especially the Sahel region, Republic of Congo, and the Lake Chad basin, where recurrent floods and droughts further impoverish communities. Conflicts often lead to the destruction of agricultural infrastructure, including irrigation systems, storage facilities, and transportation networks (Rohwerder 2017). This disruption results in reduced agricultural productivity and increased food insecurity (Lin et al. 2023). Farmers are unable to plant, tend, or harvest their crops, leading to lower yields and increased reliance on emergency food aid. Prolonged conflicts can displace farmers from their land, prompting them to seek refuge elsewhere. This displacement has led to a loss of livelihoods and traditional agricultural knowledge. This displacement interrupts the transfer of skills and practices necessary for sustainable agriculture, making it harder to implement and maintain net-zero strategies. Conflict zones often experience land degradation because of the neglected and unregulated exploitation of natural resources. Abandoned agricultural fields may revert to scrubland or be used unsustainably by displaced populations, leading to soil erosion, deforestation, and loss of biodiversity (Queiroz et al. 2014; Subedi et al. 2022). These changes increase GHG emissions and reduce the land’s capacity to act as a C sink.

Migration, both within and across borders, places additional pressure on already limited natural resources. Migrants often move to areas that are not equipped to handle sudden population increases, leading to overuse of water, land, and forests (Semenza and Ebi 2019). This overexploitation can result in environmental degradation and high GHG emissions. According to Abel et al. (2019), an increase in the number of migrants can contribute to conflict in migrant-receiving areas in many ways. This includes competition over natural and economic resources, ethnic tensions, socioeconomic tensions, and burdens on infrastructure and services (Reuveny 2007). These conflicts can further destabilize regions and disrupt agricultural activities. The resulting tension can undermine the cooperative efforts needed for sustainable land management and the implementation of net-zero agricultural practices. Migrants may use different agricultural practices that are not suited to the new environment, leading to inefficient land use and increased GHG emissions (Nguyen et al. 2023). Additionally, the need to quickly establish food security may lead to the adoption of unsustainable farming methods, such as slash-and-burn agriculture, which contributes to deforestation and soil degradation.

### Lack of awareness and misinformation on climate change and mitigation

One significant challenge in reaching this target of net-zero agriculture is the lack of awareness and understanding of climate change and its necessary mitigation strategies among the public, policymakers, and various stakeholders. Many people lack fundamental knowledge about climate change, including its causes and impacts in Africa (Ameade et al. 2023). This lack of understanding hinders informed decision-making about consumption, energy use, and support for climate policies. Despite the overwhelming scientific consensus, a significant portion of the population still denies the existence or severity of climate change. This scepticism is often fuelled by misinformation campaigns, which lead to reduced public support for necessary but potentially disruptive climate policies. According to BBC (2009), most people in African countries perceive climate change as an abstract concept that does not directly affect them, which undermines the sense of urgency required to drive meaningful and immediate action. Another aspect to consider is the economic impact as many African business organizations lack awareness of or the incentives to reduce their carbon footprint. Without consumer pressure or regulatory frameworks, profit-driven motives often take precedence over environmental considerations. Many African schools also do not normally incorporate comprehensive climate education into their curricula, leaving students without a critical understanding of the issue (Parry and Metzger 2023). This gap perpetuates ignorance and reduces the ability of future generations to effectively address climate challenges. Additionally, Baarsch and Schaeffer (2019) and Gebel et al. (2022) noted implementing measures to mitigate climate change is too costly and negatively impacts economic growth. However, this misconception overlooks the economic opportunities presented by green technologies and fails to consider the long-term costs of inaction, such as extreme weather events, health impacts, and loss of biodiversity. The rise of social media has facilitated the rapid dissemination of inaccurate and misleading information about climate science (Treen et al. 2020). Addressing this issue requires vigorous fact-checking and initiatives to advance scientific literacy.

### Competition for limited resources

Competition for resources, which encompasses land, water, financial investment, and human capital is one of the primary challenges to achieve net-zero agriculture in Africa. This competition can significantly hinder the transition to sustainable agricultural practices necessary to achieve net-zero emissions. As populations grow, the demand for food increases, leading to the expansion of agricultural land (Fitton et al. 2019). This often comes at the expense of forests and other natural ecosystems, which are vital C sinks. Balancing the need for agricultural land with conservation efforts is a major challenge.

Agriculture is the largest consumer of water in Africa, thus, sustainable water management is crucial (Tuyishimire et al. 2022). Nevertheless, the competition for water between agricultural, industrial, and domestic uses can limit the availability of this resource for implementing water-efficient and low-emission farming practices. Climate change exacerbates water scarcity through altered rainfall patterns and increased frequency of droughts. This makes efficient water management even more critical but also more challenging. Transitioning to net-zero agriculture requires significant investment in sustainable practices, technologies, and infrastructure (Climate Bond Initiative 2024). However, many African countries face financial constraints and have limited access to the necessary funding (Khan et al. 2024). Governments often need to balance investments in agriculture with other critical areas such as healthcare, education, and infrastructure development. Limited financial resources mean that agriculture may not always receive the necessary investment for sustainable transformation. Implementing net-zero agriculture also requires a workforce skilled in sustainable farming techniques, renewable energy use, and efficient resource management. However, there is often a lack of training and education in these areas, leading to a skills gap (Rikala et al. 2024). Furthermore, Abeje (2021) mentioned that rural-to-urban migration, driven by the search for better economic opportunities, can lead to labour shortages in the agricultural sector. This migration reduces the workforce available for implementing and maintaining sustainable practices.

### Low technological adoption

In Africa, achieving this ambitious goal is particularly complex because of the continent’s unique socioeconomic, environmental, and technological landscape. One of the significant barriers to this transition is the low level of technology adoption in agriculture. In many African countries, traditional farming practices dominate, and modern agricultural technologies are often limited (Janvry et al. 2017). This technology gap affects various aspects of agriculture, from crop production and soil management to water use and livestock management. Modern technologies such as precision farming, advanced irrigation systems, and data-driven decision-making tools can significantly increase productivity and sustainability. However, their adoption in Africa has been slow due to several interrelated factors.

Modern agricultural technologies require high costs of initial investment (Ruzzante et al. 2021). This is a significant hurdle as smallholder farmers, who form the backbone of African agriculture, often lack the financial resources to invest in these technologies. The costs associated with purchasing, installing, and maintaining new technologies can be prohibitive. Effective technology adoption requires robust infrastructure, including reliable energy sources, transportation networks, and communication systems. Many regions in Africa suffer from inadequate infrastructure, which hampers the implementation and efficient use of advanced agricultural technologies (Jha et al. 2020). Advanced technologies that can help achieve net-zero emissions in agriculture, such as precision farming, renewable energy systems, and advanced irrigation techniques, are often expensive and inaccessible to many smallholder farmers in Africa. The lack of infrastructure, such as reliable energy grids, transportation networks, and storage facilities, further complicates the adoption of new technologies and sustainable practices. The effective use of modern technologies often requires specialized knowledge and training. Many farmers in Africa lack access to training programmes and support services that could help them adopt and utilize these technologies effectively. To fully realize their potential benefits, even affordable technologies require proper guidance to ensure that they are utilised effectively.

Investment in agricultural research and development is relatively low in many African countries (Nin-Pratt 2021). This results in a slow rate of innovation and adaptation of technologies that are tailored to local conditions. Without localized solutions, the adoption of global technologies can be less effective or even counterproductive. In some cases, cultural preferences and traditional practices may resist the adoption of new technologies. Farmers may be hesitant to abandon tried-and-true methods, especially if they are unfamiliar with or sceptical of the benefits of new technologies. Modern technologies can also increase resilience to climate change by improving crop yields and reducing vulnerability to extreme weather events. Without these technologies, African agriculture remains more vulnerable to climate impacts, further complicating efforts to achieve net zero.

### Poor implementation of policies

There has been a proliferation of the well-intentioned policies stated above, but often the implementation of these policies is always poor and inadequate to meet the desired intentions. One of the primary issues is the lack of coordination and integration among various governmental and nongovernmental entities (Hlahla et al. 2023; Adetuyi et al. 2023). Policies designed to promote sustainable agriculture often exist in silos, with minimal collaboration between the ministries of agriculture, the environment, and finance, among others. This fragmentation leads to inconsistent policy implementation and hampers the ability to address the multifaceted nature of agricultural emissions. Effective policy implementation requires sufficient financial and human resources (Bullock and Lavis 2019). In many African countries, there is a significant gap between policy formulation and the allocation of resources necessary for their execution. Limited funding, coupled with inadequate infrastructure and technology, constrains the ability of farmers to adopt sustainable practices. Additionally, the lack of trained personnel to oversee and support the implementation of these policies further exacerbates the problem. Weak governance and institutional capacity are major impediments to the successful implementation of agricultural policies (Khanal et al. 2020). Shava and Mhlang (2023) further stated that corruption, bureaucratic inefficiencies, and a lack of accountability can derail even well-designed policies. Institutions responsible for enforcing regulations and providing support to farmers often cannot do so effectively, leading to poor compliance and suboptimal outcomes.

Weak governance and institutional capacity are major impediments to the successful implementation of agricultural policies (Nyirenda-Jere and Kazembe 2014). Political instability and frequent changes in government can lead to inconsistent and unstable policy environments (Zimmermann et al. 2009; Mvodo 2019). Policies that are initiated by one administration may be altered or abandoned by the next, creating uncertainty and discouraging long-term investment in sustainable agricultural practices. This inconsistency undermines the confidence of stakeholders in the continuity and reliability of policy support for net-zero agriculture. The effective implementation of policies requires robust monitoring and evaluation systems to track progress, identify challenges, and make necessary adjustments. However, many African countries cannot conduct comprehensive monitoring and evaluation (OECD 2021). Without reliable data and feedback mechanisms, it is not easy to assess the impact of policies and ensure that they are achieving their intended goals. Cultural and socioeconomic factors also play a significant role in the implementation of agricultural policies (Khoza et al. 2019; Pham et al. 2021). Traditional farming practices, which have been passed down through generations, can be deeply ingrained and resistant to change. Additionally, the socioeconomic conditions of many smallholder farmers, characterized by poverty and limited access to markets and credit, can hinder the adoption of new practices that require initial investment.

### Low investment in research

Research and development are critical for developing innovative solutions to increase agricultural productivity while minimizing environmental impacts. Innovative technologies and practices are essential for reducing greenhouse gas emissions and improving resource efficiency in agriculture. Low investment in research restricts the development and dissemination of such technologies. Without adequate funding, research institutions struggle to explore and implement cutting-edge solutions such as precision farming, climate-resilient crops, and efficient irrigation systems (Imran et al. 2019). Climate change poses a significant threat to agriculture in Africa, making research into climate-resilient agricultural practices crucial. Insufficient funding hampers the ability to study and develop crop varieties that can withstand extreme weather conditions, pests, and diseases. This limits farmers’ ability to adapt to changing climates, thus hindering efforts to maintain productivity while reducing emissions. Research requires robust infrastructure, including laboratories, research stations, advanced equipment, high-skilled researchers, and technicians (Seibert et al. 2024). Low investment leads to inadequate facilities and poorly maintained infrastructure, impacting the quality and scope of research outputs. Moreover, limited funding also results in fewer opportunities for capacity building and training for researchers, leading to a shortage of skilled professionals in the field.

The effective dissemination of research findings to farmers and policymakers is vital for the adoption of sustainable practices. Insufficient investment in research translates to fewer resources for extension services and knowledge transfer programmes. This gap means that even when valuable research is conducted, it often does not reach the farmers who need it most, slowing the transition to net-zero agriculture. With low domestic investment in agricultural research (Bjornlund et al. 2020), African countries often depend on external knowledge and technologies. This dependency can be problematic as imported solutions may not always suit local conditions. Building a robust, locally driven research framework is crucial for developing context-specific innovations that address unique regional challenges. Research provides the evidence base needed for sound policymaking. Insufficient investment in research leads to a lack of robust data (Christiaensen 2017) and scientific insights to inform policies aimed at achieving net-zero agriculture, e.g. accurate emission factors for GHG inventories. This can lead to ineffective or misguided policies that do not address the fundamental issues affecting the agricultural sector. Low investment in research is often a symptom of broader economic challenges. Many African countries face budgetary constraints and competing priorities, with limited funds available for agricultural research. This financial limitation hinders the ability to invest in long-term projects that are essential for sustainable agricultural development.

## Opportunities to achieve net-zero agriculture in Africa

### Opportunity for clean renewable energy

The shift to clean renewable energy in agriculture also presents substantial economic and social benefits. The contribution of renewable energy globally in 2022 was closer to around 13%, with a significant portion coming from electricity generation primarily through solar and wind power (IEA 2024). Biomass, hydropower, geothermal, solar, wind, and marine are examples of renewable energies. They have the potential to provide clean energy that may meet the net-zero targets worldwide. In Brazil, biogas and biohydrogen have been used as potential renewable energy sources (Dos Santos et al. 2025), and green electricity trading is promoted in China as an energy transition and sustainable development (Kong et al. 2025). The use of biogas for heat and electricity generation and biogas upgraded to biomethane are expected to have an increasing share in the European energy mix (Hurtig et al. 2025); the study of green technology has positively influenced renewable energy innovation in BRICS countries (Dogan et al. 2025); transition to renewable energy sources, particularly wind turbines and solar photovoltaics, is being used to achieve a climate-neutral European Union (Bessin et al. 2025); and in China, seawater source heat pumps offer promising renewable energy solutions for coastal cities, aligning with China’s carbon-neutrality goal (Wang et al. 2025). Investment in renewable energy infrastructure can stimulate local economies, create jobs, and enhance the resilience of farming communities (IRENA 2017). Farmers can enhance their economic stability and independence by lowering energy costs and increasing productivity. Renewable energy can provide a consistent and clean power supply for various agricultural operations, from irrigation systems to greenhouses. Solar-powered pumps, for example, can enable efficient water management without relying on diesel generators, thereby reducing C footprints and operating costs. African countries can utilize the sunshine to produce electricity as a source of domestic farm power to reduce the use of charcoal and firewood for cooking, especially in rural areas.

### Large population of young people

Africa’s burgeoning population of young people (Kuyoro et al. 2023) represents an unprecedented opportunity for achieving net-zero agriculture. It is estimated that the proportion of youth population (15–24 years) will decline from 19.4% in 2024 to 17.5% in 2050, but Africa will add 138 million to its youth population in this period, and by 2050 one in every three young people globally will be African (ECA 2024). The report projected that Africa’s working-age population (20–64 years) will increase from 883 million in 2024 to 1.6 billion in 2050 and constitute almost 25% of the global working-age population. This demographic shift presents a unique chance to innovate, lead, and transform agricultural practices in ways that align with environmental sustainability and economic growth. A new generation of farmers is eager to embrace sustainable practices such as agroforestry, organic farming, and conservation tillage. Education and training programmes tailored for young farmers can emphasize these methods, which contribute to C sequestration, soil health, and biodiversity. By prioritizing education on sustainable agriculture, youth can effectively contribute to achieving net-zero goals. As powerful advocates for change (Toomey et al. 2018), youth can leverage their platforms to push for policies and initiatives that support net-zero agriculture. By engaging in policymaking processes, participating in climate strikes, and using social media to raise awareness, young people can influence government actions and ensure that sustainability remains a priority in agricultural policy.

According to Hanley et al. (2020), young entrepreneurs are well positioned to start and grow green businesses focused on sustainable agriculture. Regardless of whether eco-friendly fertilizers are created, low-emission farm machinery is developed, or vertical farms are established, these startups can contribute to the net-zero vision while also driving economic development and job creation. Young people are often at the heart of community initiatives and can play a key role in educating others about the benefits of sustainable agriculture. Young researchers and scientists can drive advancements in agricultural science that contribute to net-zero goals. By focusing on research in areas such as crop genetics, soil C sequestration, and alternative proteins, new methods and technologies that help reduce agriculture’s C footprint of agriculture can be developed. Youths in Africa are increasingly connected with global networks (Adewale 2009), which could enable them to collaborate with international experts, access global resources, and participate in worldwide sustainability initiatives. Such collaborations can provide valuable insights and technologies to Africa, enhancing the continent’s ability to achieve net-zero agriculture.

### The spirit of Agenda 2063

The African Union’s Agenda 2063 represents an ambitious blueprint for the continent’s socioeconomic transformation, emphasizing sustainable development, economic growth, and environmental stewardship (AUC 2014). At its heart, Agenda 2063 envisions a future where Africa is integrated, prosperous, and resilient. As the world grapples with climate change and environmental degradation, this vision offers a crucial opportunity for Africa to achieve net-zero agriculture—a goal that harmonizes agricultural productivity with ecological balance. Agenda 2063 encourages the adoption of sustainable agricultural practices. By promoting innovations such as climate-smart agriculture, conservation tillage, and agroforestry, Africa can increase soil health, increase carbon sequestration, and reduce GHG emissions from farming activities. These practices not only help achieve net-zero goals, but also increase agricultural resilience to climate impacts.

The agenda emphasizes the importance of technology and innovation. Investments in green technologies, such as precision agriculture, renewable energy for irrigation, and efficient postharvest management, can help reduce the carbon footprint of agricultural operations. By aligning with Agenda 2063’s focus on technological advancement, African nations can lead in developing and implementing low-carbon technologies. Agenda 2063 advocates greater regional integration and cooperation. By working together, African countries can share best practices, pool resources, and develop cross-border solutions to common challenges. This collaborative approach is essential for creating a unified strategy for net-zero agriculture. Achieving net-zero agriculture aligns with Agenda 2063’s goal of ensuring food security for all Africans. Sustainable agricultural practices help maintain soil fertility, conserve water, and protect biodiversity, which are essential for long-term food production. By focusing on sustainability, Africa can achieve both food security and environmental health. The agenda highlights the need for education and capacity building. Strengthening agricultural education and training can provide farmers with knowledge about sustainable practices and climate resilience. By integrating climate education into agricultural programmes and policies, Africa can foster a new generation of farmers equipped to contribute to net-zero goals.

Agenda 2063 calls for increased investment in Africa’s development. Access to climate finance and investment from international partners can support the transition to net-zero agriculture. By aligning national and regional policies with the goals of Agenda 2063, African countries can attract funding for projects that advance sustainable agricultural practices. Climate finance has been particularly important in financing projects in Latin American countries. According to CFU (2022), a total of USD 5 billion has been approved for 550 projects in the region from multilateral climate funds since 2003, and 30 new projects were approved totalling USD 811 million in 2021, as the Green Climate Fund funded 91% of these new projects. The investments represent a small proportion in Latin America (CBI and IDB 2021), but has the potential to create between 22 and 27 million new jobs in green sectors (more than 15 million net jobs considering losses in sectors not aligned to the net zero transition) (IFC 2021). Ecuador is using climate finance to deliver the UN programme on Reducing Emissions from Deforestation and Forest Degradation (REDD+), Brazil is investing in payments in Floresta+, programme that provides incentives for environmental services to traditional communities and Indigenous peoples in the Amazon, and Costa Rica is paying for environmental programmes to women landlords and Indigenous people. Climate finance could also be useful in protecting the forest and biodiversity of West, Central, and East African countries.

### Vast arable lands in Africa

Africa’s expansive and varied landscapes possess some of the most extensive arable lands on the planet. This wealth of arable land presents a unique opportunity for the continent to transition towards net-zero agriculture. The diverse climates and soil types across Africa provide an opportunity to develop and expand sustainable crop and livestock systems tailored to local conditions. By selecting crop varieties and livestock breeds that are well adapted to local environments, farmers increase productivity and reduce environmental impacts (Eisler et al. 2014; Rivero et al. 2021a, b). Integration of diverse crop rotations and mixed farming systems can also help maintain soil fertility and reduce the risk of pest and disease outbreaks. The arable lands in Africa can be utilized for large-scale reforestation and afforestation projects (Liu et al. 2019). Planting trees on agricultural lands or integrating tree cover into farming systems can sequester substantial amounts of CO_2_, increase soil fertility, and improve water retention (Murphy 2015; Kim et al. 2016a, b). Agroforestry practices, where trees and shrubs are grown alongside crops and livestock, can create more resilient farming systems and contribute to biodiversity conservation.

### Climate-smart technologies

Precision agriculture involves the use of technology to monitor and manage field variability in crops. By utilizing sensors, GPS technology, and data analytics, farmers can optimize water, fertilizers, and pesticides. This reduces waste, lowers GHG emissions, and enhances crop yields. Climate-resilient crop varieties are genetically modified or selectively bred to withstand extreme weather conditions, such as droughts or floods. These crops have increased resistance to adverse environmental conditions, intending to maintain or increase crop yields under stress conditions (Acevedo et al. 2020; Kopeć 2024), maintain yields, and reduce the need for additional inputs that contribute to greenhouse gas emissions.

Conservative agriculture which focuses on minimizing soil disturbance, maintaining soil cover, and rotating crops could be adopted. These practices improve soil health and increase the ability to store carbon. Utilizing renewable energy sources, such as solar or wind, to power agricultural operations reduces reliance on fossil fuels and decreases greenhouse gas emissions. Smart irrigation systems use sensors and automated controls to apply water precisely when and where it is needed (Obaideen et al. 2022). This helps conserve water and reduces the energy required for irrigation.

## Conclusions, future directions, and recommendations

Africa’s shift to net zero is critical for economic growth due to population growth, urbanization, and economic transformation, which will exert pressure on the traditional high-emitting agricultural sector. African countries are particularly vulnerable to climate-related risks (e.g. rainfall agriculture, and drought), which threaten food security. Emissions from AFOLU may increase due to unsustainable land use changes and management strategies, including deforestation, increased fertilizer use, and expanding agriculture for the supply, especially rice production. While there are numerous challenges facing the continent’s transition to net-zero agriculture, there are also several opportunities. Addressing these challenges can provide hope and encourage countries within the continent to transition to net-zero agriculture. There are significant research gaps related to the interactions among land use, climate, soils, and ecosystems. This leads to a poor understanding of Africa’s contribution to GHG emissions. African countries, where AFOLU activities represent a large portion of national economies, are particularly at risk from climate change. They may benefit considerably from receiving climate funding for strategies that link GHG reduction to resilience, food security, and rural development goals. The current focus of African countries on strengthening the resilience of economic systems from the damaging effects of climate change, if planned holistically, can address GHG emissions reduction in agriculture, which is the main source of livelihoods on the continent and a key producer of overall emissions. Although the current understanding of GHG emissions in SSA is limited, the region’s potential as both a source and a sink of GHGs is substantial. By addressing these issues holistically and engaging stakeholders at all levels, Africa can move towards a more sustainable agricultural future that balances resource use with environmental stewardship. These challenges and limitations can be addressed through the following suggested pathways to achieve net-zero agriculture in Africa.

- **Mobilizing climate finance:** Leveraging additional resources from international donors, the private sector, and innovative financing mechanisms will help to expand successful initiatives. Securing climate finance is crucial to support the implementation of climate action plans and strategies across all levels. ClimDev-Africa should pursue innovative financing options, such as green bonds and public–private partnerships, to mobilize resources for climate action. Additionally, strengthening collaborations with international climate funds, such as the Green Climate Fund, can further enhance funding opportunities.
- **Foster regional cooperation and knowledge sharing:** Enhanced regional collaboration and knowledge exchange will help to tackle transboundary climate challenges, including shared water resources and migratory patterns. This can be achieved by establishing regional centres of excellence to foster research, innovation, and knowledge sharing in climate science and adaptation strategies. Additionally, strengthening South–South cooperation will enable the exchange of best practices and technologies for effective climate adaptation and mitigation.
- **Strengthening climate information services:** Africa should prioritize investment in modern climate data collection and analysis tools to enhance the quality and accessibility of climate information. This includes upgrading meteorological infrastructure with advanced weather stations, satellite technology, and remote sensing systems to improve data accuracy and reliability. Additionally, capacity-building initiatives should focus on training local meteorologists, climatologists, and other key stakeholders to interpret and apply climate data effectively. Providing information in local languages can further ensure that communities fully understand and utilize it. Moreover, the development of user-friendly platforms, such as mobile apps and web portals, should be encouraged to disseminate climate information efficiently. These platforms should be tailored to reach farmers—particularly youth and women—as well as policymakers and other end users, enabling informed decision-making and enhancing resilience to climate change.
- **Promote inclusive policies and climate resilience programmes: **Africa should ensure that climate policies are inclusive and address the needs of vulnerable groups, such as women, youth, civil societies, and Indigenous communities. These policies should align with broader development objectives and maintain coherence across various sectors and levels of government. A key focus should be on developing specific policies aimed at achieving net-zero agriculture. Strengthening the capacity of national and regional institutions to effectively implement and enforce climate policies and regulations is essential for meeting net-zero targets. All stakeholders must integrate climate change considerations into national and regional development plans, policies, and strategies. Encouraging agricultural practices that boost productivity, enhance resilience, and reduce greenhouse gas emissions is critical. Furthermore, infrastructure designed to withstand extreme weather events and adapt to changing climate conditions should be prioritized and developed across the continent.
- **Promoting gender-responsive climate action:** It is important to mainstream climate policies and programmes that consider the different needs, roles, and vulnerabilities of men and women. Women constitute a large percentage of the agricultural workforce in Africa, and supporting their participation in climate decision-making, as well as providing them with access to resources and technologies for climate adaptation, will be vital for achieving net-zero agriculture on the continent. This focus is particularly crucial in addressing the unique impacts of climate change on women, especially in rural and marginalized communities.
- **Fostering innovation and technology transfer:** The continent requires robust research and innovation hubs, as well as universities, to spearhead cutting-edge research on climate change impacts, adaptation, and mitigation strategies tailored to its unique challenges. Research in climate-smart agriculture in areas of conservation agriculture (minimal soil disturbance, crop rotation, organic mulching, etc.) improved crop and climate-adaptive crop varieties, and efficient water management (drip irrigation, rainwater harvesting, and soil moisture conservation) could be vital tools for climate resilience on the continent. Additionally, research in renewable energy and adoption in areas such as solar power, biogas and biofuels, wind, and hydro energy could facilitate reduced emissions from agriculture on the continents. Facilitating the transfer of climate-friendly technologies from developed countries to Africa, particularly adaptation technologies and climate-related startups, could significantly accelerate the transition to net-zero agriculture.
- **Addressing climate-induced migration and displacement:** The continent must establish early warning systems to anticipate and reduce the impacts of climate-induced migration and displacement. It is essential to create alternative livelihood opportunities for communities vulnerable to displacement caused by climate change. This should be supported by robust policies and frameworks to effectively manage climate-related migration and safeguard the rights and well-being of displaced populations.

## Supplementary Information

Below is the link to the electronic supplementary material.

Supplementary file1 (DOCX 35 KB)
