# Supplementary material for: Achieving net-zero agriculture in Africa: perspective on policies, challenges, and opportunities
Source: Sustain Sci. 2025 Mar 17;20(3):1117–37. doi: 10.1007/s11625-025-01666-y (PMC12033123; doi:10.1007/s11625-025-01666-y)
Supplement: Supplementary file 1 — Supplementary file1 (DOCX 35 KB) [file 11625_2025_1666_MOESM1_ESM.docx]

**Supplementary materials**

**Achieving net-zero agriculture in Africa: perspective on policies, challenges, and opportunities**

**Tersur Theophilus Akpensuen^1,2^* and M. Jordana Rivero^1^**

*^1^Net-zero and Resilient Farming, Rothamsted Research, North Wyke, EX20 2SB, Okehampton, United Kingdom*

*^2^Faculty of Agriculture, University of Jos. 930001, Jos, Nigeria*

**Corresponding Author’s email address:* [*theo.akpensuen@rothamsted.ac.uk*](mailto:theo.akpensuen@rothamsted.ac.uk)

#### **Table S1:** The three-tiered African climate change negotiation structure (Watson, 2022)

| **Tier** | **NAME** | **Description** |
| --- | --- | --- |
| First | Committee of African Heads of State and Government on Climate Change (CAHOSCC) (Makina, 2016)  (<https://au.int/en/agenda2063/overview>) | CAHOSCC was established in 2009 by the AU Assembly of Heads of State and Government to spearhead the African Common Position on Climate Change and to ensure that Africa speaks with one voice in global climate change negotiations. CAHOSCC comprises Algeria, the Democratic Republic of Congo, the Republic of Congo, Equatorial Guinea, Ethiopia, Kenya, Mali, Mauritius, Mozambique, Nigeria, South Africa, Uganda, and the Chairperson of the AU Commission. This is the highest political tier in the African climate change negotiation structure. It is mandated to spearhead the African Common Position on Climate Change and its key messages and ensure that Africa speaks with one voice in global climate change negotiations |
| Second | African Ministerial Conference on the Environment (AMCEN). (Makina, 2013) <https://www.unep.org/regions/africa/african-ministerial-conference-environment/about-amcen> | Established in 1985 following a conference of African ministers of environment, AMCEN is mandated to provide advocacy for environmental protection in Africa, to ensure that basic human needs are met adequately and in a sustainable manner, to ensure that social and economic development is realised at all levels, and to ensure that agricultural activities and practices meet the food security needs of the region. |
| Third | African Group of Negotiators (AGN). (Watson, 2022) <https://www.unep.org/regions/africa/african-ministerial-conference-environment> | The AGN consists of climate change negotiators from every African country. It was established at COP1 in 1995, it is an alliance of African Member States that **represents the interests of the region in international climate change negotiations**, with a common and unified voice. One country is selected to chair the group for two years. During the COP and intersessional negotiations, the AGN, represented by the relevant lead coordinator, is the technical body that participates in negotiations. It receives guidance from the African Ministers of Environment (AMCEN), the CAHOSCC, and the African Union Assembly. |

**S2:** Continental level policies, frameworks, and action plans to mitigate climate change.

| **S/no.** | **Policies/frameworks/Action plans** | **Summary description of aim and objectives** | **Timeframe** |
| --- | --- | --- | --- |
| **African Union Continental Policies** | | | |
| **1** | Agenda 2063: Africa, We Want *(*[*https://au.int/en/agenda2063/overview*](https://au.int/en/agenda2063/overview)*)* | Agenda 2063 advocates for united efforts, self-reliance, and Africa financing its climate-smart, inclusive, and people-driven development while speaking with one voice in global forums. This philosophy underpins the Africa Climate Change Strategy. The Strategy was developed by reviewing the 2014 Draft Continental Climate Change Strategy, the 2015 Paris Agreement, the United Nations Sustainable Development Goals, the Africa Programme of Action on Disaster Risk Reduction, the Sendai Framework, and various national and regional climate change frameworks. Feedback from key stakeholders, Regional Economic Communities, academia, the African Ministerial Conference on the Environment, and civil society was incorporated to create a comprehensive and updated African climate change strategy. It is the continent’s 50-year strategic framework that aims to deliver on its goal for inclusive and sustainable development and is a concrete manifestation of the Pan-African drive for unity, self-determination, freedom, progress, and collective prosperity pursued under Pan-Africanism and African Renaissance. Recognizing the dynamic and complex interrelationships in the world, the Africa Climate Change Strategy aims to be a living document that evolves with global, continental, regional, and national developments. | 2013– 2063 |
| **2** | Africa Climate Change strategy *(*[*https://au.int/sites/default/files/documents/41959-doc-CC_Strategy_and_Action_Plan_2022-2032_08_02_23_Single_Print_Ready.pdf*](https://au.int/sites/default/files/documents/41959-doc-CC_Strategy_and_Action_Plan_2022-2032_08_02_23_Single_Print_Ready.pdf)*)* | The main goal of the Africa Climate Change Strategy is to achieve the Agenda 2063 Vision by enhancing the resilience of the African continent to climate change impacts. The Logical Framework serves as a roadmap, outlining how the Africa Climate Change Strategy will reach its specific objective: attaining SDG 13, which calls for urgent action to combat climate change. This will be achieved through coordinated adaptation and mitigation responses across Africa. The strategy envisions five key results: 1 Strengthened institutional capacities to implement climate change strategies; 2. Harmonization of climate change strategies; 3. A unified African voice on climate change issues; 4 Enhanced resilience and reduced vulnerability; 5 Increased access to climate finance. This strategy is designed for the African Union, Regional Economic Communities in Africa, and their respective organs and agencies, as well as Member States' departments and institutions responsible for climate change. Non-governmental organizations, partners, and the private sector are encouraged to align their climate change strategies and programs with the Africa Climate Change Strategy to ensure coherent climate action across the continent. | 2020 - 2030 |
| **3** | Comprehensive African Agricultural Development Programme (CAADP). <https://caadp.org/> | The Comprehensive African Agricultural Development Programme (CAADP) is a key initiative under Agenda 2063, aiming to eliminate hunger and reduce poverty in Africa through agriculture-led development. African governments, under CAADP, committed to allocating at least 10% of national budgets to agriculture and achieving at least 6% annual agricultural growth. The program reduces poverty and malnutrition, increases productivity and farm incomes, and promotes sustainable agricultural practices. Additionally, CAADP enhances resilience to climate variability by developing disaster preparedness policies, early warning systems, and social safety nets. It serves as Africa’s policy framework for agricultural transformation, wealth creation, food security, nutrition, and economic growth. | 2003 – 2025 |
| **4** | African Union Sustainable Forest Management Framework  <https://afforum.org/publication/the-sustainable-forest-management-framework-for-africa-2020-2030/> | African forests are vital for maintaining environmental quality and stability on both regional and global scales. They provide essential ecosystem services and are key to achieving several UN Sustainable Development Goals, including SDG1 (no poverty), SDG2 (zero hunger), SDG13 (climate action), SDG14 (life below water), and SDG15 (life on land). Sustainable Forest Management supports global objectives set by the United Nations Forum on Forests and other related frameworks. Covering 624 million hectares, which is 20.6% of Africa’s land area and 15.6% of the world’s forest cover, African forests play a unique role in achieving the goals of Agenda 2063. This agenda envisions a prosperous continent that drives its development through sustainable and long-term resource management. | 2020 – 2030 |
| **5** | Integrated African Strategy on Meteorology (weather and climate services)  <https://amcomet.wmo.int/sites/default/files/field/doc/pages/wmo_amcomet_strategy_en_0.pdf> | The Strategy aims to integrate weather, water, and climate services into national and regional development frameworks for sustainable development in Africa, focusing on poverty reduction, climate change adaptation, and disaster risk reduction. Its objective is to enhance cooperation among African countries and strengthen government capabilities to provide these essential services, with National Meteorological and Hydrological Services (NMHSs) playing a critical role. The Strategy aligns with Agenda 2063, the Paris Agreement, the Sendai Framework, and the UN SDGs. It aims to create coordinated mechanisms for strategic direction, helping streamline policies to develop adequate weather, water, and climate services at all levels. | 2021 - 2030 |
| **6** | Great Green Wall Initiative  <https://www.unccd.int/our-work/ggwi> | The Great Green Wall (GGW) was an African-led initiative to plant trees over an 8,000-kilometer stretch across the Sahel to combat desertification. Proposed in the 1980s and renewed in the early 2000s, it aimed to moderate temperatures, reduce wind speeds, prevent soil erosion, and increase local humidity for agriculture. Launched in 2007 and set for completion in 2030, the project aimed to be the largest manmade structure, costing $8 billion and funded by 21 Sahelian countries, the World Bank, the EU, and the UN. It was designed to absorb 250 million tonnes of CO_2_ annually, create 10 million green jobs, increase rainfall, recharge water tables, and rejuvenate biodiversity. The GGW aimed to restore 100 million hectares of degraded land and was implemented across 22 African countries, fostering economic opportunities, food security, and climate resilience. With leadership from the African Union Commission and the Pan-African Agency of the Great Green Wall, over USD 14 billion was raised to support this transformative initiative. | 2007 - 2030 |
| **7** | African Forest Landscape Restoration Initiative (AFR100). <https://afr100.org/> | AFR100 addresses the African Union's mandate to restore 100 million hectares of degraded land by 2030, following the political declaration endorsed by the African Union in October 2015 for the Africa Resilient Landscapes Initiative (ARLI). It supports the African Landscapes Action Plan (ALAP) and the broader Climate Change, Biodiversity, and Land Degradation (LDBA) program. AFR100 helps achieve domestic restoration and sustainable development goals, the Bonn Challenge, and the New York Declaration on Forests, among other targets. The initiative contributes to the Sustainable Development Goals (SDGs) and the Paris Climate Agreement, building on the progress made through the Terr Africa Partnership and related landscape restoration efforts. | 2015 – 2030 |
| **8** | Initiative for the Adaptation of African Agriculture to Climate Change.  [Initiative for the Adaptation of African Agriculture to climate change \| Department of Economic and Social Affairs (un.org)](https://sdgs.un.org/partnerships/initiative-adaptation-african-agriculture-climate-change) | Launched at COP22 in Marrakesh in November 2016, the "Initiative for the Adaptation of African Agriculture to Climate Change" (AAA Initiative) aims to enhance food security in Africa, improve conditions for vulnerable farmers, and promote rural employment by encouraging climate change adaptation practices. It supports African countries in implementing their Nationally Determined Contributions (NDCs) under the Paris Agreement. The initiative focuses on soil management, agricultural water management, and climate risk management, utilizing UNFCCC-recommended instruments like technology transfer, appropriate agricultural policies, and "bankable" projects. Backed by 36 African countries and recognized by the African Union, the AAA Initiative channels climate finance and supports project implementation to help African agriculture adapt to climate change. Governed by the Annual Conference of African Ministers of Agriculture, it is hosted by the AAA Initiative Foundation in Rabat, Morocco. | 2016 – 2022 |
| **9** | AU Green Recovery Action Plan (AU GRAP)  <https://au.int/sites/default/files/documents/40790-doc-AU_Green_Recovery_Action_Plan_ENGLISH1.pdf> | The Green Recovery Action Plan addresses the dual challenges of COVID-19 recovery and climate change by prioritizing key areas such as climate finance, renewable energy, resilient agriculture, resilient cities, land use, and biodiversity. The objectives are to enhance collaboration on shared priorities supporting the African Union’s goals for sustainable and green recovery from COVID-19 and to support the vision of a prosperous, secure, inclusive, and innovative future for Africa. | 2021 – 2027 |
| **10** | Pan-African Action Agenda on Ecosystem Restoration for Increased Resilience  <https://www.cbd.int/doc/c/274b/80e7/34d341167178fe08effd0900/cop-14-afr-hls-04-final-en.pdf> | The agenda outlines policy measures, strategic actions, cooperation mechanisms, and practical steps to enhance land and ecosystem restoration across Africa. The Pan-African Action Agenda on Ecosystem Restoration for Increased Resilience focuses on advancing these efforts with four main activities: (a) assessing opportunities for ecosystem restoration, (b) improving the institutional framework for ecosystem restoration, (c) planning and implementing restoration activities, and (d) monitoring, evaluating, and disseminating results. | 2016 – 2030 |
| **11** | Science, Technology, Innovation Strategy for Africa <https://au.int/en/documents/20200625/science-technology-and-innovation-strategy-africa-2024> | The strategy aimed to expedite the transition of African countries toward innovation-driven and knowledge-based economies. This goal was to be realized by enhancing Africa's readiness in science, technology, and innovation and by implementing targeted policies and programs that addressed societal needs comprehensively and sustainably. Developed during a pivotal phase when the African Union was creating its long-term Agenda 2063, the STISA-2024 represented the first of a series of ten-year phased strategies designed to meet the growing demand for science, technology, and innovation. It focused on critical sectors including agriculture, energy, environment, health, infrastructure development, mining, security, and water. The strategy was grounded in six key priority areas aligned with the AU Vision: Eradicating Hunger and Achieving Food Security; Preventing and Controlling Diseases; Enhancing Communication (both physical and intellectual mobility); Protecting Space; Fostering Social Cohesion; and Creating Wealth. Additionally, it outlined four essential pillars for success: building or upgrading research infrastructures; enhancing professional and technical skills; promoting entrepreneurship and innovation; and creating a supportive environment for STI development across Africa. Continental, regional, and national programs were to be designed and synchronized to ensure that their strategic orientations and pillars worked together to achieve the desired developmental outcomes as effectively as possible. | 2014 – 2024 |

**S3:** Regional climate Centres policies, frameworks, and action plans to mitigate climate change in agriculture.

| **S/no** | **Policies/frameworks/Action plans** | **Description** | **Timeframe** |
| --- | --- | --- | --- |
| **1** | East Africa Community (EAC) Climate Change Policy.  <https://www.rema.gov.rw/rema_doc/Climate%20change/EAC%20Climate%20Change%20Policy_April%202011.pdf> | The Policy's overarching goal is to foster sustainable development in the EAC region by implementing harmonized and coordinated regional strategies, programs, and actions to address climate change. It aims to guide EAC Partner States and other stakeholders in executing collective measures to tackle the impacts and causes of climate change through adaptation and mitigation, ensuring sustainable social and economic development. This Policy offers an integrated, harmonized, multi-sectoral framework for responding to climate change in the EAC Partner States through both adaptation and mitigation efforts. | 2020 - 2030 |
| **2** | Economic Community of West African States (ECOWAS) Strategic Programme on Reducing Vulnerability and Adapting to Climate Change.  <https://ecowas.int/wp-content/uploads/2022/09/ECOWAS-Regional-Climate-Strategy_FINAL.pdf> | A strategy to reduce vulnerability to climate change and build the resilience of affected communities is essential. Given the severity of the anticipated impacts, "Acting Together" within a framework of regional solidarity is crucial for reducing the region's vulnerability and collectively addressing the borderless risks induced by climate change. Building on its past experience with the strategic program for reducing vulnerability and adapting to climate change, funded by Sweden, ECOWAS is enhancing its action framework by systematically integrating climate change impacts into its actions and directives. The vision is to create a community resilient to the effects and impacts of climate change while capitalizing on associated economic opportunities for long-term, low-carbon, sustainable development. The general objective of ECOWAS' regional climate strategy is to support Member States in overcoming the challenge of combating climate change, particularly in fulfilling their commitments under the Paris Agreement. | 2020 - 2030 |
| **3** | South African Development Community (SADC) Climate Change Strategy and Action Plan (CCSAP) <https://www.sadc.int/sites/default/files/2021-11/SADC_Climate_Change_Strategy_and_Action_Plan-English.pdf> | The vision is to establish a climate-resilient and low-carbon regional economy. The SADC Climate Change Strategy and Action Plan outlines harmonized and coordinated regional and national actions to address climate change impacts in alignment with global and continental goals. It emphasizes enhanced adaptation to diverse and gender-specific vulnerabilities while supporting appropriate mitigation actions for sustainable development. The strategy, guiding the Climate Change Programme from 2015 to 2030, provides a framework for short, medium, and long-term climate change adaptation and mitigation programs and projects. It is divided into three categories: (i) Climate Change Adaptation, (ii) Climate Change Mitigation, and (iii) Means of Implementation and Monitoring and Evaluation. The strategic objectives are to reduce vulnerability and manage climate-related risks through effective adaptation programs, promote the reduction of greenhouse gas emissions considering member states' capabilities, and enhance the region's capacity to mobilize resources, access technology, and build capacity for adaptation and mitigation actions | 2015 - 2030 |
| **4** | Common Market for East and Southern Africa (COMESA) Strategy on Climate Change <https://www.comesa.int/wp-content/uploads/2022/09/220908_Climate-Change-and-Inclusive-Growth.pdf> | The COMESA Regional Resilience Implementation Plan and Resource Mobilization Strategy is nearing completion following final review by experts in climate change, resilience, disaster risk reduction and management, drawn from 14 Member States, Regional Economic Communities (RECs) and the African Union Commission. The Strategy defines the main parameters for an effective climate change response in the COMESA region that builds resilient adaptive capacities and unlocks the benefits of the mitigation potential of the region. | **2020-2030** |
| **5** | IGAD Climate Prediction and Applications Centre (ICPAC)  <https://www.icpac.net/> | ICPAC is a specialized institution of the Inter-Governmental Authority on Development (IGAD). Its objectives include enhancing the technical capacity of both producers and users of climate information, developing a proactive, timely, and comprehensive system for disseminating information and products, and expanding the knowledge base within the sub-region to support informed decision-making by fostering a clearer understanding of climate and climate-related processes. IGAD Member States are Djibouti, Ethiopia, Eritrea, Kenya, Somalia, Sudan, South Sudan, and Uganda. IGAD’s Climate Center and provides climate services and supports national institutions to improve their service delivery in areas such as climate forecasting, monitoring of food security, crop, rangelands, pests, forests, natural habitats, or supporting climate change adaptation and mitigation | **2021** |
| **6** | ECCAS Action Plan For Implementation of the Central Africa Gender Responsive Regional Strategy for Risk Prevention, Disaster Management and Climate Change Adaptation. <https://atjhub.csvr.org.za/economic-community-of-central-african-states/> | The Economic Community of Central African States (ECCAS) action plan aims to help member states develop mechanisms to manage disasters, such as droughts and flooding, with a focus on gender. This approach is important because disasters often affect women and men differently, and women need to be actively involved in risk management strategies, as they are crucial agents in this field yet underrepresented. Section 2, Priority 4 of the action plan highlights the importance of "building back better" in terms of "recovery, rehabilitation, and reconstruction" after disasters. This includes integrating women's voices in post-disaster coordination efforts and addressing gender-specific needs | **2020 – 2030** |
| **7** | The Arab Maghreb Union (AMU). <https://au.int/en/recs/uma> | The Arab Maghreb Union (AMU), formed in 1989 by Algeria, Libya, Mauritania, Morocco, and Tunisia, aimed to enhance regional cooperation and development. The member states committed to coordinating policies for sustainable development across all sectors, as formalized by the Treaty and the Marrakesh Summit's Solemn Declaration. The AMU's goals include strengthening fraternal ties, promoting societal progress and rights, preserving peace with justice, adopting common policies, and gradually enabling the free movement of people, services, goods, and capital among the member nations. | **1889** |
| **8** | Community of Sahel-Saharan States (CEN-SAS). <https://au.int/en/recs/censad> | The Community of Sahel-Saharan States (CEN-SAD) was established in 1998 to promote regional economic integration and cooperation among its member states. It became a regional economic community in 2000 and gained observer status at the UN General Assembly. CEN-SAD's objectives include forming an economic union, removing barriers to unity, facilitating free movement, enhancing transportation and telecommunications, and coordinating educational systems. In 2013, CEN-SAD endorsed a restructuring and introduced a revised Treaty focusing on regional security and sustainable development, which will take effect after ratification by fifteen member states, of which thirteen have ratified. The revised organizational structure includes various councils and institutions such as the Conference of Heads of State/Government, the permanent Peace and Security Council, and the Sahel-Sharan Bank for Investment and Trade. | **1998** |

**References**

Watson C (2022) Options for Embedding Article 2.1 c in the New Collective Quantiﬁed Goal on Climate Finance. ODI; 2022. <http://cdn-odi-production.s3.amazonaws.com/media/documents/ODI_Working_paper_Options_for_embedding_Articles_2.1c_in_NQCGs_C0cAJnW.pdf> Accessed 24 August 2024.
